# Optimizing preparation of low-NaCl protein gels from goose meat and understanding synergistic effects of pH/NaCl in improving gel characteristics

**DOI:** 10.1016/j.fochx.2024.101333

**Published:** 2024-03-30

**Authors:** Xinxin Yan, Mingpeng Xie, Zhonghai Hu, Jingjun Li, Haibo Zheng, Ningning Xie, Zongyuan Zhen

**Affiliations:** a College of Food Engineering, Anhui Science and Technology University, Chuzhou 233100, China; b Lu'an Longxiang Gourmet Poultry Co., Ltd., Lu'an 237400, China; c Institute of Agro-product Science and Technology, Anhui Academy of Agricultural Sciences, Hefei 230031, China; d Anhui Engineering Laboratory for Functional Microorganisms and Fermented Foods, Hefei 230031, China; e The Institute of Functional Agriculture (Food) Science and Technology at Yangtze River Delta (iFAST), Chuzhou 239000, China; f Anhui Provincial Key Laboratory of Functional Agriculture and Functional Food, Chuzhou 233100, China

**Keywords:** Gel properties, Salt-soluble protein, Low sodium, Response surface methodology, Synergistic analysis

## Abstract

This study explored the feasibility of partially substituting NaCl with MgCl_2_ in preparing gel products from goose meat. Furthermore, the effects of synergistic interaction between different pH levels and NaCl concentrations on the structure and characteristics of the gels were explored by analyzing their secondary structure, microstructure, and water-distribution properties. The results showed that NaCl could be partially substituted by MgCl_2_, with the optimal preparation conditions: NaCl (0.83 mol/L), pH (7.3), MgCl_2_ (0.04 mol/L), heating temperature (79 °C), heating time (20 min), and solid-liquid ratio (1:3). Furthermore, the pH had a more significant impact on the gels' structure and characteristics than did NaCl concentration. Thus, our optimized method can reduce the usage of NaCl in the gel products while at the same time improving the characteristics of gel products under low-NaCl conditions by lowering pH, laying a solid theoretical foundation for producing low-NaCl protein gel products from goose meat.

## Highlights

• An optimal approach in preparing salt-soluble protein gels from goose meat.
• MgCl_2_ can partially replace NaCl to prepare low-NaCl protein gels from goose meat.
• Adjusting pH can counteract negative effect of low-NaCl on quality of protein gel.

## Graphical abstract

**Figure:**
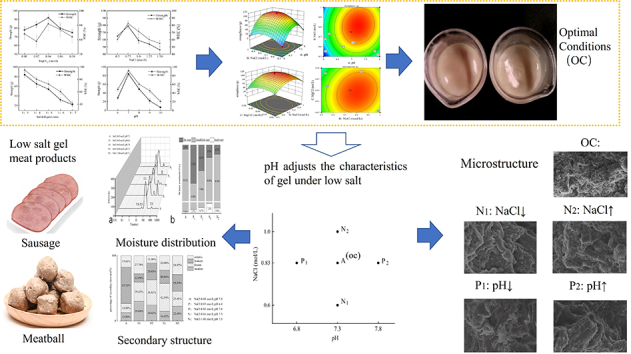

## 1 Introduction

China is the world's largest producer of goose meat, with an annual production of ∼2.52 million tons (Huo et al., 2021). The Landaise goose is famous as a breed for foie gras. Actually, its slaughter rate and muscle nutritional value are also high among commercially used goose breeds (Yan et al., 2022), so the utilizing of meat of Landaise can increase the added value of foie gras. Goose meat is nutrient-rich and contains high levels of essential amino acids and unsaturated fatty acids (Yan et al., 2022). While goose meat is protein-rich, its fibers are relatively coarse (Weng et al., 2021); thus, it is generally processed into meat-paste foods like emulsified sausages and meatballs. The quality of these meat paste products is mainly affected by their texture, water retention capacity, and adhesion properties, characteristics which are largely influenced by the gelation and emulsification properties of muscle protein. Salt-soluble proteins, accounting for 70–90% of muscle protein, are a main component and play a key role in these processes, particularly in thermal gelation in minced meat products (Wang, Zhou, & Chen, 2022). Extraction of salt-soluble protein from goose meat is harder than from meat of other species because of the relatively crude fiber of goose meat (Weng et al., 2021). Therefore, the preparation of protein gel from goose meat is more challenging compared with other traditional methods for meat in some other species.

The effectiveness of muscle protein solubilization in meat product processing depends on a combination of NaCl and minimal amounts of complex phosphates. However, high‑sodium products are detrimental to human health, increasing risk of chronic diseases such as hypertension or cardiovascular diseases among consumers (Brand, Visser, Schoonees, & Naude, 2022). In contrast, MgCl_2_ has more similar salty flavor to NaCl compared to sodium-substitutes like calcium., while it has higher ionic strength than sodium-substitutes like potassium. At the same time, Mg^2+^ is beneficial in lowering blood pressure (Pelczyńska, Moszak, & Bogdański, 2022). Previous studies have shown that MgCl_2_ can be used to partially replace sodium salts to prepare pork (Ge, Han, Zheng, Zhao, & Sun, 2020) and chicken (Xiong & Brekke, 2007) salt-soluble protein gel products.

In addition, NaCl content can be further reduced by adjusting the synergistic effect between NaCl and different conditions (e.g., ultrasound, sodium bicarbonate, and soy protein) (Zhang, Guo, & Shi, 2023). pH (Yang et al., 2020) and NaCl content (Zhu et al., 2022) are usually considered the main factors affecting the properties of protein gels from meat. For instance, pH and NaCl play dominant roles in the formation of different meat gels (Lee & Chin, 2020; Xue, Pei, Wen, Chin, & Hu, 2023). Other than reducing the NaCl content in these protein gel products, it is also necessary to ensure that the preparation approach does not affect the gel's characteristics and tastes. Currently, studies on salt-soluble protein gel products are mainly focused on pork, beef (Widyastuti, Rosyidi, Radiati, & Purwadi, 2020), and chicken (Sun, Wu, Xu, & Li, 2012), and the research on goose meat is limited. Thus, here we explored how to prepare low-NaCl protein gels from goose meat by incorporating MgCl_2_ and utilized the response surface method to investigate how parameters such as time, temperature, MgCl_2_, pH, and NaCl concentration, can affect the properties of protein gels. Meanwhile, based on the optimal parameters, this manuscript explains how the synergistic interaction between pH and NaCl can affect the proteins' microstructure, secondary structure, and water distribution properties. With our findings, it is possible to reduce NaCl content in protein gel and mitigate the adverse impacts of low sodium content on gel properties through adjusting the pH. Thus, our study offers a theoretical framework for the production of high-quality low-NaCl protein gel products from goose meat.

## 2 Materials and methods

### 2.1 Experimental materials

The goose meat utilized in our experiment was sourced from 80-day-old Landaise geese, randomly selected from the same batch raised at Longxiang Gourmet King Poultry Company Limited, Lu’an (Anhui, China). The carcasses were maintained at −18 °C freezer until later use. All other chemical reagents are purchased from McLean Biochemical Co. and are of analytical grade.

### 2.2 Sample preparation

#### 2.2.1 Raw material handling

The frozen goose carcasses were thawed at 4 °C, and breast meat was collected from 12 Landaise geese and cut evenly. After trimming the visible connective and fatty tissues, the meat was cubed into uniform parts measuring ∼2 cm^3^, which were then evenly divided into portions weighing around 30 g each for subsequent salt-soluble protein extraction processes (Yan et al., 2024).

#### 2.2.2 Extraction of salt-soluble proteins

Each piece of goose meat (i.e., ∼30 g) was added into 90 mL solution [0.75 mol/L NaCl, 0.4 mol/L of MgCl_2_] (i.e., at a volume vs. weight ratio of 1:3). The meat was homogenized using a high-speed tissue masher and further processed by a high-shear homogenizing emulsifier at a speed of 15,000 rpm/min until a meat slurry was formed. The pH of the mixture was then adjusted to 6.8 using either 0.2 mol/L HCl or NaOH solution with a computerized bench-top acidometer. The homogenate was refrigerated at 4 °C for one day, and then forced through a single layer of gauze and centrifuged at 13,000 r/min for 15 min using a high-speed centrifuge (H4-20KR). The supernatant was further filtered with a double-layered gauze (16 mesh) to get salt-soluble protein solution (Smyth, McCord, & O'Neill, 1998).

#### 2.2.3 Preparation of salt-soluble protein gels

Briefly, for texture analysis, 6 mL of salt-soluble protein mixture was added into a 10 mL beaker (25 mm in radius and 35 mm in height). For water retention analysis, the same volume of salt-soluble protein mixture was added into a 10 mL centrifuge tube. The sample solution in both the beaker and tube was heated in a water bath, whose temperature increased at a rate of 1 °C per minute; when reaching 80 °C, the incubation was maintained for 25 min. The heated samples were cooled to ambient temperature. The resulting protein gels were subsequently stored in a 4 °C freezer (Chen et al., 2016).

### 2.3 Experimental design

#### 2.3.1 Optimization of parameters

##### 2.3.1.1 Single factor test

Single-factor experiments were conducted to assess the influence of various parameters, including MgCl_2_ concentration (0.04 mol/L), NaCl concentration (0.75 mol/L), heating temperature (80 °C), time (20 min), solid-liquid ratio (1:3), and pH (7), on the gel strength and water-holding capacity (WHC) of salt-soluble protein gels from goose meat. The pH levels tested were 6, 7, 8, 9, and 10; the NaCl concentrations tested were 0.5, 0.75, 1.0, 1.25, and 1.5 mol/L; the MgCl_2_ concentrations tested were 0, 0.02, 0.04, 0.06, and 0.08 mol/L; the solid-liquid ratios tested were 1:3, 1:4, 1:5, 1:6, and 1:7; the heating temperatures tested were 60, 70, 80, 90, and 100 °C; the time lengths for the constant heating temperatures tested were 5, 10, 20, 30, and 40 min. Gels were prepared and evaluated for their WHC and gel strength under the above conditions.

##### 2.3.1.2 Response surface methodology (RSM) test

The parameters that most significantly affected the WHC and gel strength of the protein gels were determined based on results from the single-factor experiments which utilized the Box-Behnken design (i.e. RSM). A total of 4 factors were selected for subsequent experiments. The coding of the selected factor levels is shown in Table 1.

**Table 1:** Box-Behnken design of RSM test.

Factor	Level		
	−1	0	1
A: pH	6	7	8
B: NaCl (mol/L)	0.5	0.75	1
C: MgCl_2_ (mol/L)	0.2	0.4	0.6
D: Temperature (°C)	70	80	90

#### 2.3.2 Synergistic analysis of effects of pH and NaCl on the structure and properties of the gels based on the RSM result

As shown in Fig. 3, the samples were categorized into 5 groups based on the results of the RSM test. Group A represents the most optimal condition from these results. Groups P_1_ and P_2_ represent the low and high pH conditions, respectively. Groups N_1_ and N_2_ represent the low and high NaCl conditions, respectively. The rest of the conditions were the same for all these five groups, all are optimal conditions based on results from response surface experiments, i.e., MgCl_2_ concentration of 0.04 mol/L, a solid-liquid ratio of 1:3, heating temperature of 79 °C, and heat time of 20 min. The measurements, including secondary structure, moisture distribution, and microstructure, in samples from the five groups, were compared to determine what combination of NaCl concentration and pH level has a maximal effect on protein gel characteristics.

### 2.4 Detection methods

#### 2.4.1 Determination of gel strength

A texture meter (Stable MicroSystem TA-XT-PLUS) was used to measure the protein gel's gel strength, with probe type being P/0.5, a measuring speed being 1.0 mm/s, a post-testing speed being 1.00 mm/s, a measuring distance being 4 mm, the trigger type being set at automatic, a trigger time being 5 s, a trigger force being 5.0 g, a data acquisition rate being 200.00 Hz (Zhu et al., 2022). Each test was repeated for 3 times to obtain accurate results.

#### 2.4.2 Detection of WHC

Briefly, before gel preparation, the tube was weighed on an electronic analytical balance and recorded as m. Post gel preparation, the tube which contained the salt-soluble protein gel was weighed again and recorded as m_1_. Next, the gels were centrifuged at 3000 rpm for five minutes; the supernatant was removed, and the residual water was removed using paper filters. The tubes were weighed again and recorded as m_2_. For accuracy, the tests were conducted at 4 °C, with three replicates for each sample. The gel's WHC was calculated using formula (1) (Yan et al., 2024).

(1) $$\mathrm{WHC}\;\left(\%\right)=\frac{\left(m2-m\right)}{\left(m1-m\right)}\times 100$$

#### 2.4.3 Determination of moisture distribution

Freshly prepared protein gels were maintained in a 4 °C refrigerator for 12 h prior to moisture distribution analysis using a low-field nuclear magnetic analyzer (Neumay NMI21-040H-I). The samples were sectioned into 10 mm cubes, each with identical mass; the sample cubes were each placed in a glass vial with the vial mouth diameter <25 mm for NMR measurement. Key parameters of the T_2_ measurements included the following: resonance frequency = 20 MHz, TW = 4500 ms, TE = 1.2 ms, NS = 8, and NECH = 8000. The spin-spin relaxation time (T_2_) was determined by the Carr Purcell Mbom Gill (GPMC) sequence. The resulting curves were then inverted to identify the T_2_ components (T_2b_, T_22_, and T_23_) and their proportions (PT_2b_, PT_22_, and PT_23_) were recorded (Guo et al., 2019).

#### 2.4.4 Determination of secondary structure

Raman spectroscopy (HORIBA XploRA PLUS) was used to identify the proteins' secondary structures. Briefly, 1.5 g of the sample was prepared on a slide and analyzed under the following settings: a laser wavelength of 532 nm, laser power of 100 mW, a scanning range of 400–3600 cm^−1^, a spectral resolution of 2.0 cm^−1^; three scans per sample, 60 exposures, data collection at 1 cm^−1^ intervals, and a scanning speed of 120 cm/min. Upon completion of the test, normalization was performed using phenylalanine (1003 cm^−1^) as the reference. Analysis of the protein amide I band (1600– 1700 cm^−1^) was conducted using Peakfit 4.12. This procedure involved baseline correction, deconvolution, and second derivative fitting. Following Alix et al., the relative content of the myofibrillar protein secondary structure was determined by calculating the area of each sub-peak (Feng et al., 2023).

#### 2.4.5 Determination of microstructure

The protein gels were cut into squares (0.5 cm × 0.5 cm × 0.3 cm) and fixed in 10 mL of 2.5% glutaraldehyde solution for 24 h. Subsequently, the gel squares were rinsed three times with 0.2 M PBS (8.1 mM Na_2_HPO_4_, 1.9 mM NaH_2_PO_4_, 0.1 M NaCl, pH 7.0, 4 °C), each time for 10 min. Next, they were dehydrated in an ethanol gradient series of 50%, 60%, 70%, 80%, 90%, and 100%, with each step lasting 15 min and repeated three times. Then, the gel squares were soaked in a mixture of 100% ethanol and tert-butanol (1:1 in ratio) for 15 min, followed by freeze-drying for 36 h. The gel's microstructures were then examined under a scanning electron microscope (ZEISS EVO-18) at a magnification of 2000× and a voltage setting of 15.0 kV (Zhao, Zhou, & Zhang, 2019).

### 2.5 Data analysis

Data for each variable were collected in triplicate, compiled, and analyzed. The results are visualized using the Origin 2021 software. One-way ANOVA was used for multiple comparisons between groups using the SPSS20 software. The Box-Behnken response surface analysis was conducted using the DesignExpert12 software. Data from the one-way tests were further processed to obtain the regression equations, variances, and multifactorial interaction effects on the protein gel's tensile strength and WHC. In all cases, a threshold of *p* < 0.05 indicates statistical significance.

## 3 Results

### 3.1 Optimization of various parameters for extracting protein gels

#### 3.1.1 Single factor test

The optimal conditions identified through the single-factor experiments were as follows: MgCl_2_ 0.04 mol/L, NaCl 0.75 mol/L, pH 7, solid-liquid ratio 1:3, heating temperature 80 °C, and heating time 20 min.

**Fig. 1:**
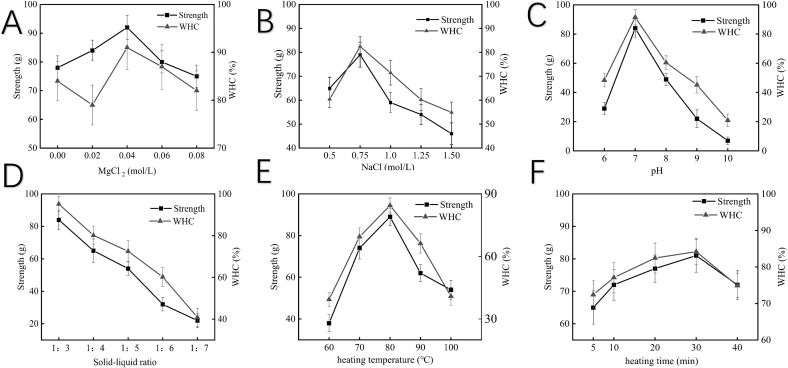
Effects of different MgCl_2_ concentrations, NaCl concentrations, pH, solid-liquid ratios, and heating temperature and time on gel strength and WHC of salt-soluble protein gels from goose meat.

As shown in Fig. 1A, the addition of a small quantity of MgCl_2_ enhanced both the gel strength and WHC, displaying an initial increase followed by a decrease as the MgCl_2_ concentration rose. The peak gel strength was achieved at a MgCl_2_ concentration of 0.04 mol/L. Thus, concentrations of 0.02–0.06 mol/L were selected for subsequent studies (Table 1). As depicted in Fig. 1B, both the gel strength and WHC rose initially and then decreased as the content of NaCl rose. Specifically, the peak strength and WHC of the gels were achieved at a NaCl concentration of 0.75 mol/L. Thus, concentrations of 0.5–1 mol/L were selected for subsequent studies (Table 1). As illustrated in Fig. 1C, as the pH rose, both the gel strength and WHC gradually increased, peaked at pH 7, and then gradually decreased. Thus, pH 6–8 was chosen for the subsequent study (Table 1). As displayed in Fig. 1D, a decrease in both the strength and WHC was observed when the solid-liquid ratio ranged between 1:3 and 1:5. Considering its influence on gel formation and the centrifugation process, a solid-liquid ratio of 1:3 was selected. As displayed in Fig. 1E, both the strength and WHC increased until they reached the peak when the heating temperature was at 80 °C. Thus, 70–90 °C was chosen for the subsequent study (Table 1). As Fig. 1F shows, the WHC initially rose then subsequently dropped with heating time increased, whereas the strength gradually increased without significant change. Optimal properties were achieved when the heating time ranged between 20 and 40 min. Considering the practical operations, heating time of 20 min was chosen.

#### 3.1.2 Response surface methodology (RSM) test

**Fig. 2:**
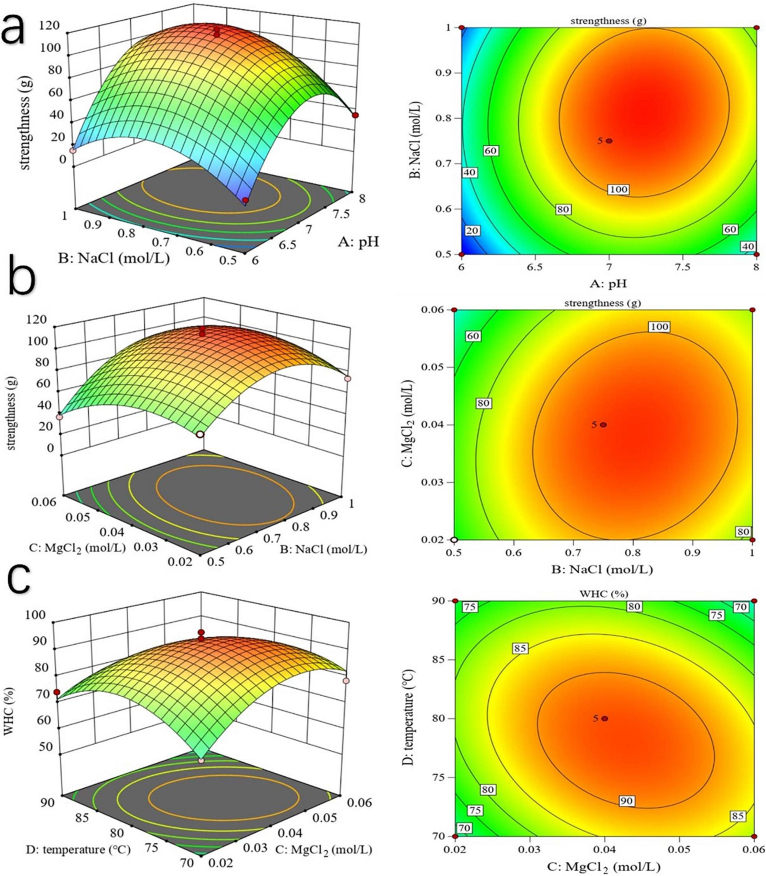
Response surface diagram illustrating the significant interactions between parameters of the salt-soluble protein gels from goose meat. Three interactions are significant (*p* < 0.05) according RSM analysis. a, the influence of the interaction between pH and NaCl concentration on gel strength; b, the influence of the interaction between MgCl_2_ concentration and heating temperature on gel strength; c, the influence of the interaction between MgCl_2_ concentration and NaCl concentration on WHC.

In the response surface analysis, the following parameters were selected: pH (A), NaCl concentration (B), MgCl_2_ concentration (C), and heating temperature (D). Table 1 outlines the coding for each factor level. The analysis revealed that the impact of these factors on the strength of the gels, ranked by the magnitude of their F-values, was A (204.32) > B (75.19) > D (23.37) > C (9.61). Significant interactions were observed between pH and NaCl concentration and between NaCl concentration and MgCl_2_ concentration (Fig. 2). The fitted regression equation was as follows: strength (Y1) = 112.40 + 22.67A + 13.75B − 4.92C + 7.67D + 6.5AB − 2.5AC + AD +7BC − 4.75BD − 1.75CD − 50.95A^2^–30.57B^2^–17.57C^2^–17.70D^2^. The analysis also revealed that the impact of these factors on the WHC, ranked by their F-values, was B (65.55) > A (34.45) > D (11.11) > C (4.09). The WHC was significantly influenced by the interaction between pH and NaCl concentration (Fig. 2). The fitted regression equation was as follows: water retention (Y2) = 92.99 + 5.84A + 8.11B + 2.01C − 3.31D + 2.09AB + 1.39AC − 1.56AD + 2.56BC − 3.14BD − 5.17CD − 11.75A^2^–13.45B^2^–10.42C^2^–10.99D^2^.

**Fig. 3:**
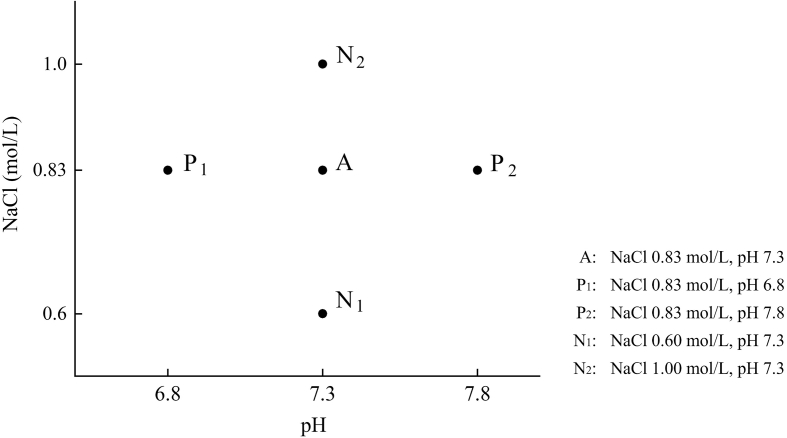
Synergistic effects between pH level and NaCl concentration on the characteristics of salt-soluble protein gels. Group A represents the most optimal condition from the result of RSM test. Groups P1 and P2 represent the low and high pH conditions, respectively. Groups N1 and N2 represent the low and high NaCl conditions, respectively.

**Fig. 4:**
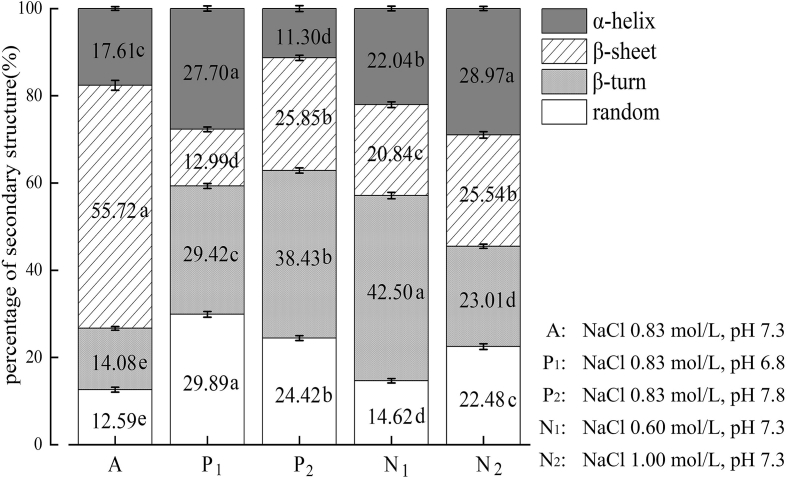
Differences in the secondary structure of the protein gels under different pH/NaCl combinations. Group A has the highest content of β-sheet; Group P_1_ has the lowest content of β-sheet; Group P_2_ has the lowest α-helix content; Group N_1_ has the highest β-turning content; while Group N_2_ has a relatively even proportion of the four components. The results are presented as mean ± SD (each variable in triplicate). Different lowercase letters located in the same legends indicate significant differences among treatments (p < 0.05).

**Fig. 5:**
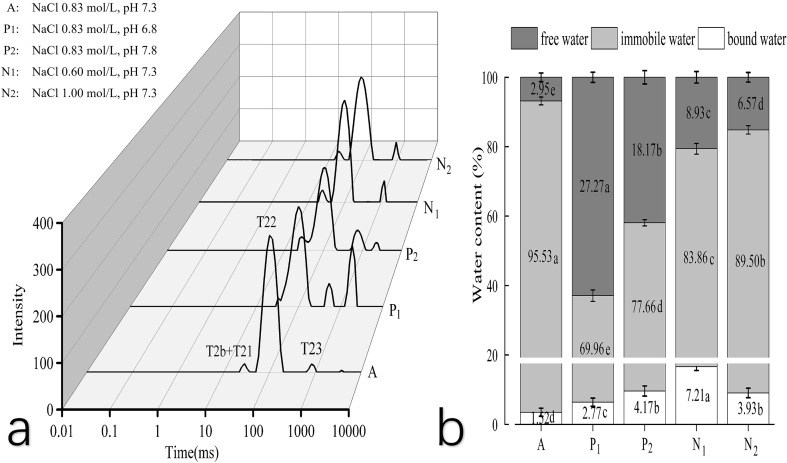
Variations in transverse relaxation time T2 and water distribution in salt-soluble protein gels from goose meat under different pH/NaCl conditions. Four different peaks, i.e., T2b (0.1–1 ms), T21 (1–10 ms), T22 (10–200 ms), and T23 (>200 ms), were observed. T2b and T21 represent bound water; T22 represents immobile water (a); and T23 represents free water. Group A demonstrates a shorter relaxation time (a) and is associated with a higher percentage of immobile water (b). The results are presented as mean ± SD (each variable in triplicate). Different lowercase letters located in the same legends indicate significant differences among treatments (p < 0.05).

**Fig. 6:**
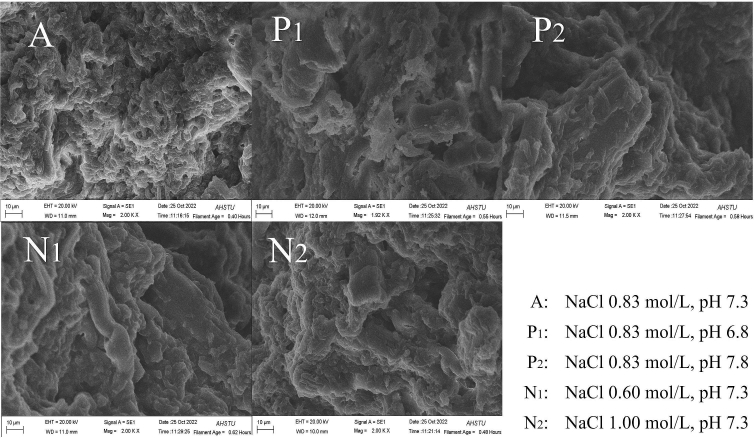
Microstructure of the protein gels under different conditions. Group A exhibits a relatively dense and uniform structure with small pore sizes; Group P_1_ contains larger gel voids; Group P_2_ has obvious structural defects; Group N_1_ has a less dense gel network structure; and Group N_2_ displays a relatively discontinuous gel structure. Gel void structure in Group N_1_ is denser than those in Groups P_1_ and P_2_.

Based on above equations, the theoretical optimal conditions for producing low-salt protein gels from goose meat were as follows: NaCl (0.83 mol/L), pH (7.3), MgCl_2_ (0.04 mol/L), heating temperature (79 °C), heating time (20 min), solid-liquid ratio (1:3). Based on these theoretical conditions, the maximum strength and WHC of the low-NaCl protein gels from goose meat were 115.92 g and 95.55%, respectively. To validate these theoretical predictions, a validation test was carried out under the theoretical conditions. The observed gel strength was 113 ± 6 g and the WHC was 96.15 ± 2.54%. The discrepancies between the theoretical and actual values were not significant (*p* > 0.05), demonstrating the reliability and efficacy of the optimized parameters determined by the model.

### 3.2 Different pH and NaCl concentration combinations affected structure and characteristics of the salt-soluble protein gels

#### 3.2.1 Secondary structure of the salt-soluble protein gels from goose meat

Raman spectroscopy, a straightforward and risk-free technique, is broadly employed to determine proteins' secondary structure (Ma, Sun, Pan, Cao, & Dang, 2022). As shown in Fig. 4, group A exhibited the highest β-sheet content compared to the other groups (*p* < 0.05). Lower α-helix and higher β-sheet contents in the gels (p < 0.05) indicate improved gel characteristics, suggesting the formation of a more organized 3-D protein network and increased hydrogen bonding between protein molecules(Han, Liu, Cao, Yue, & Shao, 2023). Group P_1_, characterized by the lowest β-sheet content (p < 0.05), suggests that low pH significantly affects the secondary structure of protein gels. Specifically, increasing the pH to control conditions induced an increase in ordered structures and a decrease in disordered structures within the gel (p < 0.05). Group P_2_, with the highest proportions of β-turn and irregular curl (p < 0.05), suggests that the alkaline condition causes mutual repulsion between neighboring molecules, leading to larger particle sizes that are not conducive to protein cross-linking (Mao et al., 2023). Group N_1_, containing the highest content of β-turn and a slightly higher proportion of α-helix structure with lower β-sheet contents (p < 0.05), suggests that the low NaCl concentrations do not favor the unfolding of α-helix structure. Despite this, the β-sheet structure content was higher in this group compared with that in groups P_1_ and P_2_. Group N_2_ (*p* < 0.05), containing an enhanced transformation from β-sheet structure to β-turn structure with irregular curling, suggests that high NaCl concentrations tend to drive the gel structure toward a more disordered state.

#### 3.2.2 Moisture distribution status of the salt-soluble protein gels from goose meat

Low-field NMR is an effective tool for analyzing water mobility and distribution within protein gels (Wang et al., 2021). The relaxation time, T2, serves as an indicator of how tightly water molecules are bound to the substrate. A shorter T2 signifies stronger hydrogen bonding and less water mobility, while a longer T2 indicates a weaker bonding of water molecules to macromolecules. Specifically, T2b and T21 represent bound water, T22 represents water with limited mobility, and T23 represents free water (Lu et al., 2023). In the T2 spectra for salt-soluble protein gels from goose meat under different conditions (Fig. 5a), Group A displayed the shortest T22 relaxation time and the highest peak area. In contrast, Groups P_1_ and P_2_ exhibited two peaks for free water and a lower T22 peak area. Groups N_1_ and N_2_ not only had lower T22 peak areas but also longer T22 relaxation times, suggesting a weakened ability for these two groups to retain water molecules effectively. As shown in Fig. 5a, Group A features three peaks with high relative strength and stable morphology, while Groups P_1_ and P_2_ present two peaks for free water with lower peak areas.

The percentage of water in various states within the salt-soluble protein gels from goose meat is quantified in Fig. 5b, based on the sum of peak areas in the corresponding relaxation time spectra. Group A exhibited a significantly higher proportion of immobile water, while a significantly lower proportion of bound and free water, compared to other groups (*p* < 0.05). This suggests a transformation of bound and free water into immobile water, resulting in tighter molecule binding and enhanced water stability within the gels. Notably, Group N_1_ displayed the highest proportion of immobile water, exceeding the levels in both Groups P_1_ and P_2_ (Fig. 5b, p < 0.05), which had optimal NaCl concentration but varied pH levels. Conversely, Group P_1_, characterized by a high pH, exhibited the highest percentage of free water (p < 0.05), suggesting the strong effect of pH on the gel's WHC.

#### 3.2.3 Microstructural analysis of salt-soluble protein gels from goose meat

As a control group, the Group A with most optimal conditions showed a highly organized, dense, and homogenous gel network with small pore sizes (Fig. 6). This intricate network suggests stronger intermolecular interactions within the gel, corroborating the optimization results that indicated peak strength under these conditions. In comparison, Group P_1_ displayed large particles on the gel's surface, leading to a looser network organization, which is consistent with the result in Group P_1_ showing the lowest percentage of immobile water (Fig. 5b). In Group P_2_, a lamellar structure was observed, and the network exhibited irregularities and a loosening in its cross-linking state, leading to fractures in the gel and a compromised structure, finally resulting in reduced capacity to hold bound water. Group N_1_ displayed pores of varying sizes and the connecting fibers in the network are denser. Group N_2_ was marked by obvious surface pores, and the protein network structure was incomplete.

#### 3.2.4 Synergistic analysis of effects of pH level and NaCl concentration on the structure and properties of the gels

As shown in Table 2, the water distribution and secondary structures of salt-soluble protein gels varied under different pH and NaCl concentration combinations. Group N1 (low salt + optimal pH) exhibited superior gel properties compared to both Group P1 (optimal NaCl concentration + low pH) and Group P2 (optimal NaCl concentration + high pH).

**Table 2:** Impacts of various circumstances on the characteristics of salt-soluble protein gel.

Different group	NaCl		pH		Stacked Gel Properties	
	Variations	Gelatinous	Variations	Gelatinous	Water distribution	Secondary structure
P_1_ → N_2_	+	−	+	+	+	+
N_1_ → P_2_	+	+	+	−	−	−
N_2_ → P_2_	−	+	+	−	−	n/a
N_1_ → P_1_	+	+	−	−	−	−

+ indicates enhancement, − indicates weakening, and n/a indicates no significant difference (p < 0.05).

## 4 Discussions

### 4.1 Optimized parameters and their synergistic effects

Currently, there are no existing reports on optimizing the preparation of salt-soluble protein gels from goose meat. Compared to those for chicken (Smyth et al., 1998) and pork (Shi, Zheng, Liu, Gao, & Shao, 2021), the existing procedure for preparing protein gels from goose meat requires more NaCl and higher heating temperature to achieve better gel textural properties. This could be attributed possibly to the unique inherent features of goose meat. The study from Xing et al. (Xing et al., 2017) also demonstrates that protein gels' water retention capacity can be improved at NaCl concentrations similar to those of our current study. Interestingly, the pH levels required for our process are higher than those needed for chicken (Foegeding, 2006) but lower than those for pork (Choi et al., 2011). Nonetheless, both pH levels yielded gels from chicken and pork with stable properties.

While pH had a stronger effect on gel strength, NaCl concentration was more influential on WHC. These observations align well with previous studies showing the dominant roles of pH and NaCl in meat gel formation. Molecular dynamics modeling suggests that pH and NaCl concentration influence protein structure in distinct ways (Bai et al., 2023). Specifically, pH exerts a greater effect on particle size, amino acid spatial organization, and protein conformation, while NaCl primarily affects protein solubilization by altering ionic strength in the solution (Li, Zhang, Lu, & Kang, 2021).

The interaction between pH and NaCl concentration exerts a significant influence on the strength of protein gels (*p* < 0.05). Gel strength is primarily determined by the type and quantity of cross-links in salt-soluble proteins under heat treatment. During gel formation, the ambient pH affects the charge balance of the amino acid terminal chains, subsequently influencing protein interactions (Sun et al., 2019). On the other hand, higher NaCl concentration can lower the isoelectric point of these proteins, increasing their net negative charge, and thereby enhancing their solubility and processability (Zhu et al., 2022). The combined effects of these factors elevate protein surface hydrophobicity while simultaneously improving solubility, which promotes gel formation. Thus, in low NaCl concentrations, altering the pH could be a feasible strategy for the preparation of salt-soluble protein gels.

The synergistic effects between MgCl_2_ concentration and heating temperature also significantly affect the gel's WHC. As the temperature rises, heat-treated proteins denature, unfold, and expose their internal hydrophobic groups, thereby increasing their affinity for water molecules and ensuring stability during centrifugation (Lu, Liang, Zhang, & Wen, 2022). Moreover, divalent Mg^2+^ ions and exposed sulfhydryl groups can form disulfide bonds, producing cross-linking structures, favoring protein aggregation, enhancing interactions between proteins, and between protein and water, and consequently, enhancing the protein network and gel strength.

The synergistic effect between MgCl_2_ and NaCl concentrations was also relatively strong. NaCl promotes protein solubility and increases protein surface hydrophobicity, while Mg^2+^ ions can promote the generation of disulfide bonds from sulfhydryl groups through exchange or oxidation reactions, improving the gels' WHC (Ge et al., 2020).

### 4.2 The structures and characteristics of the protein gels: The synergistic effect of pH and NaCl

Response surface tests in this study revealed some variability in the effects of pH and NaCl on gel properties. However, it is unclear which factor, pH or NACL, has a greater impact on the gel. Therefore, based on optimized results of response surface test, this study also explores synergistic effects of pH and NaCl concentration on gel characteristics of salt-soluble proteins from goose muscle.

A robust gel structure is typically marked by a high content of β-sheet, and the quantity of immobile water can serve as an indicator of strength of protein-water binding (Wang et al., 2021). Therefore, these two factors were employed to assess the strength of gels in this study. Compared to Group A, the percentage of β-sheet (Fig. 4) and immobile water (Fig. 5b) in Groups P_1_, P_2_, N_1,_ and N_2_ showed varying degrees of decline.

As shown in Fig. 4, Fig. 5, Fig. 6, Groups A, P_1,_ and P_2_ displayed distinct gel characteristics across varying pH levels under a constant NaCl concentration. A comparison between the immobile water percentage (Fig. 5b) and β-sheet (Fig. 4) showed the following trend: A > P_2_ > P_1_ (*p* < 0.05). pH affects protein-molecule interactions by influencing charge states of protein surface and electrostatic repulsive forces (Dumetz, Chockla, Kaler, & Lenhoff, 2008). Compared to Group A (pH 7.3), Group P_1_ (pH 6.8) is situated closer to the protein's isoelectric point. This proximity results in a decline in the myofibrillar protein's negative charge, leading to reduced electrostatic repulsion. Consequently, protein aggregations are more prone to flocculation and precipitation, thereby weakening the interactions between water and protein. In contrast, Group P_2_ exhibits a higher pH level (pH 7.8). This increased pH results in an excessive negative charge on the protein and a greater exposure of hydrophobic groups during structural unfolding, further intensifying the electrostatic repulsion between proteins and making it unattainable to form a stable gel network structure. Furthermore, Group P_1_ displayed lower levels of immobile water percentage (Fig. 5b) and β-sheet (Fig. 4) compared to Group P_2_ (p < 0.05). This suggests that a decrease in pH from the optimal level exerts a more pronounced effect on protein gel characteristics than an increase does. Similar change patterns are also observed in previous studies on pork (Zhang, Puolanne, & Ertbjerg, 2021) and duck (Ndi & Brekke, 2006) under varying pH conditions.

As illustrated in Fig. 4, Fig. 5, Fig. 6, Groups A, N_1_ and N_2_ exhibited varying gel characteristics of salt-soluble proteins under different NaCl concentrations while at constant pH. When comparing immobile water percentage (Fig. 5b) and β-sheet (Fig. 4), a trend is observed as follows: Group A > Group N_2_ > Group N_1_ (p < 0.05). Compared to Group A (0.83 mol/L), a very low NaCl concentration in Group N_1_ (1.00 mol/L) resulted in diminished extraction and solubilization of myosin and actin within the salt-soluble proteins, and made the proteins less susceptible to thermal denaturation, thereby adversely affecting their capacity to retain water. In contrast, compared to Group A, a very high concentration of NaCl concentration in Group N_2_ could lead to the “salting out” effect among proteins, weakening electrostatic repulsion and enhancing peptide chain aggregation and protein precipitation. This can further hinder the exposure of hydrophilic groups on the amino acid side chains, thereby reducing the proteins' ability to retain water and increasing the proportion of free water (Zhuang et al., 2019). An elevated ionic strength also weakens hydrogen and ionic bonding within the gel, disrupting the tertiary structure of myosin and leading to its changed conformation and a progressively disordered gel structure. Microstructural analyses of this study further confirmed that high salt content is not conducive to forming an ideal protein gel. Moreover, Group N_1_ displayed lower immobile water and β-sheet content compared to Group N_2_ (Fig. 5b, p < 0.05), suggesting that reducing NaCl concentrations below the optimal level has a more pronounced effect on the protein gel's characteristics than increasing the NaCl concentration (Cao et al., 2023).

In Group A with the most optimal conditions, the intermolecular electrostatic repulsive force reaches equilibrium. This allows for high solubility and stability of the salt-soluble proteins, minimizing the aggregation between protein molecules, leading to an optimal lattice structure. Regarding microstructures (Fig. 6), the gap in the gel of P_1_ was larger than that of P_2_, and the gap in the gel of N_1_ was larger than that of N_2_, indicating that the protein gel network structure was weakly cross-linked with each other, and the structure was relatively loose. Of note, the microstates of gels in the P_1_ and P_2_ groups were more affected than those of N_1_ and N_2_ groups, indicating that pH has a stronger effect in destroying the gel network structure than NaCl.

Based on the analyses and subgroup comparisons (Table 2), it is evident that a decrease, rather than an increase, in pH and NaCl concentration from optimal levels has a more significant impact on the characteristics of salt-soluble protein gels. That is, the structure and characteristics of the protein gels prepared under low-salt conditions can be improved by adjusting the pH level. This observation is particularly important because it indicates that pH has a greater influence on the gel characteristics than NaCl concentration. As shown in Table 2, varying pH levels and NaCl concentrations were associated with distinct gel characteristics. The observed combined effects of both factors were always consistent with the corresponding changes resulting from pH, further supporting the findings of Xue et al. (Xue et al., 2023), which suggested that pH has a more substantial influence on the gels' characteristics than NaCl concentration.

## 5 Conclusion

In this study, MgCl_2_ has been utilized to partially substitute NaCl in the production of salt-soluble protein gels from goose meat. The optimal processing conditions were determined through a series of experiments as follows: NaCl (0.83 mol/L), pH (7.3), MgCl_2_ (0.04 mol/L), heating temperature (79 °C), heating time (20 min), and solid-liquid ratio (1:3). Under these parameters, the gel strength and WHC were measured at 113 ± 6 g and 96.15 ± 2.54%, respectively, which was well aligned with the model predictions; moreover, the internal microstructure was more stable and the WHC was enhanced. Furthermore, our comparative analysis under various pH and NaCl concentrations revealed that both factors contribute to the formation of a dense gel network structure. This is achieved mainly through transforming α-helix to β-sheet structures, and by increasing the proportion of immobile water within the gels. The synergistic effect analysis showed that pH levels and NaCl concentrations lower than those in the optimal conditions can achieve comparable gel characteristics, indicating that adjusting pH levels can further decrease the usage of NaCl. In conclusion, MgCl_2_ can be used to partially substitute NaCl to prepare low-NaCl protein gels from goose meat, and pH adjustment can enhance the structure and characteristics of protein gels under low-NaCl conditions. Our study thus laid a theoretical foundation for the production of premium low-sodium protein gels from goose meat. Further research may focus on exploring the impact of different MgCl_2_ to NaCl ratios on the textural and nutritional attributes of goose meat gels. Additionally, investigating the consumer acceptability of these low-NaCl gels and their potential applications in various culinary contexts could provide valuable insights for the food industry.

## CRediT authorship contribution statement

**Xinxin Yan:** Writing – review & editing, Writing – original draft, Visualization, Software, Methodology, Investigation, Formal analysis, Data curation, Conceptualization. **Mingpeng Xie:** Investigation, Formal analysis, Data curation. **Zhonghai Hu:** Resources, Project administration, Funding acquisition. **Jingjun Li:** Resources, Project administration. **Haibo Zheng:** Resources, Project administration. **Ningning Xie:** Validation, Investigation. **Zongyuan Zhen:** Writing – review & editing, Writing – original draft, Validation, Supervision, Resources, Project administration, Methodology, Investigation, Funding acquisition, Formal analysis, Conceptualization.

## Declaration of competing interest

The authors declare that they have no known competing financial interests or personal relationships that could have appeared to influence the work reported in this paper.

## Data Availability

Data will be made available on request.

## Appendix A Supplementary data

ANOVA of response surface quadratic model for water retention and gel hardness of protein gel from goose meat. Image 1
