# Supplementary material for: Optimizing preparation of low-NaCl protein gels from goose meat and understanding synergistic effects of pH/NaCl in improving gel characteristics
Source: Food Chem X. 2024 Mar 30;22:101333. doi: 10.1016/j.fochx.2024.101333 (PMC11002545; doi:10.1016/j.fochx.2024.101333)
Supplement: Supplementary file 1 — ANOVA of response surface quadratic model for water retention and gel hardness of protein gel from goose meat. [file mmc1.docx]

**Table 3** Analysis of variance results of water retention regression model

| source | square sum | degrees of freedom | mean square | F | P |
| --- | --- | --- | --- | --- | --- |
| Model | 3876.04 | 14 | 276.86 | 23.34 | < 0.0001 |
| A-pH | 409.83 | 1 | 409.83 | 34.54 | < 0.0001*** |
| B-NaCl | 789.51 | 1 | 789.51 | 66.55 | < 0.0001*** |
| C-MgCl_2_ | 48.53 | 1 | 48.53 | 4.09 | 0.0626 |
| D- temperature | 131.82 | 1 | 131.82 | 11.11 | 0.0049** |
| AB | 17.39 | 1 | 17.39 | 1.47 | 0.2461 |
| AC | 7.72 | 1 | 7.72 | 0.651 | 0.4333 |
| AD | 9.77 | 1 | 9.77 | 0.8231 | 0.3796 |
| BC | 26.24 | 1 | 26.24 | 2.21 | 0.1591 |
| BD | 39.5 | 1 | 39.5 | 3.33 | 0.0895 |
| CD | 106.83 | 1 | 106.83 | 9 | 0.0095** |
| A² | 895.05 | 1 | 895.05 | 75.44 | < 0.0001*** |
| B² | 1172.66 | 1 | 1172.66 | 98.84 | < 0.0001*** |
| C² | 704.91 | 1 | 704.91 | 59.42 | < 0.0001*** |
| D² | 784.12 | 1 | 784.12 | 66.09 | < 0.0001*** |
| residual | 166.09 | 14 | 11.86 |  |  |
| incoherent | 145.43 | 10 | 14.54 | 2.82 | 0.1652 |
| difference | 20.66 | 4 | 5.17 |  |  |
| composite | 4042.13 | 28 |  |  |  |
| R^2^=0.9589. R^2^ADJ=0.9178 | | | | | |

Note: "*" represents significant (p < 0.05); "* *" represents significant (p < 0.01); "* * *" represents extremely significant (p < 0.001).

**Table 4** Analysis of variance of regression equation for gel hardness

| source | square sum | degrees of freedom | mean square | F | P |
| --- | --- | --- | --- | --- | --- |
| Model | 29977.41 | 14 | 2141.24 | 70.96 | < 0.0001*** |
| A-pH | 6165.33 | 1 | 6165.33 | 204.32 | < 0.0001*** |
| B-NaCl | 2268.75 | 1 | 2268.75 | 75.19 | < 0.0001*** |
| C-MgCl_2_ | 290.08 | 1 | 290.08 | 9.61 | 0.0078** |
| D- temperature | 705.33 | 1 | 705.33 | 23.37 | 0.0003** |
| AB | 169 | 1 | 169 | 5.6 | 0.0329* |
| AC | 25 | 1 | 25 | 0.8285 | 0.3781 |
| AD | 4 | 1 | 4 | 0.1326 | 0.7212 |
| BC | 196 | 1 | 196 | 6.5 | 0.0232* |
| BD | 90.25 | 1 | 90.25 | 2.99 | 0.1057 |
| CD | 12.25 | 1 | 12.25 | 0.406 | 0.5343 |
| A² | 16838.29 | 1 | 16838.29 | 558.02 | < 0.0001 |
| B² | 6063.77 | 1 | 6063.77 | 200.95 | < 0.0001 |
| C² | 2003.55 | 1 | 2003.55 | 66.4 | < 0.0001 |
| D² | 2032.15 | 1 | 2032.15 | 67.35 | < 0.0001 |
| residual | 422.45 | 14 | 30.18 |  |  |
| incoherent | 341.25 | 10 | 34.13 | 1.68 | 0.3255 |
| difference | 81.2 | 4 | 20.3 |  |  |
| composite | 30399.86 | 28 |  |  |  |
| R^2^=0.9861. R^2^ADJ=0.9722 | | | | | |

Note: "*" represents significant (p < 0.05); "* *" represents significant (p < 0.01); "* * *" represents extremely significant (p < 0.001).
